# Acute phenylcapsaicin supplementation improves CrossFit® performance: a randomized, triple-blind, placebo-controlled crossover trial

**DOI:** 10.1080/15502783.2026.2615274

**Published:** 2026-01-14

**Authors:** Alejandro R. Triviño, Carlos Díaz-Romero, Juan J. Martin-Olmedo, Pablo Jimenez-Martinez, Carlos Alix-Fages, Magdalena Cwiklinska, Daniela Pérez, David Funes Pol, Lucas Jurado-Fasoli

**Affiliations:** a Department of Chemical and Pharmaceutical Technology Engineering, University of La Laguna, La Laguna, Spain; b Department of Physiology, Faculty of Medicine, Sport and Health University Research Institute (iMUDS), University of Granada, Granada, Spain; c Research Group in Prevention and Health in Exercise and Sport (PHES), University of Valencia, Valencia, Spain; d Department of Health Research, Icen Cognis, Santa Cruz de Tenerife, Spain; e Applied Biomechanics and Sport Technology Research Group, Autonomous University of Madrid, Madrid, Spain; f Neonatology and UMIP Service, University Hospital Nuestra Señora de La Candelaria, Santa Cruz de Tenerife, Spain; g Departamento de Ciencias Médicas Básicas, Universidad de La Laguna EVOPRED- Universidad Europea de Canarias, Spain; h Centro de Investigación Biomédica en Red Fisiopatología de la Obesidad y Nutrición (CIBERobn), Instituto de Salud Carlos III, Madrid, Spain; i Instituto de Investigación Biosanitaria ibs.Granada, Granada, Spain

**Keywords:** ergogenic aid, capsaicinoid, cross-training, performance, nutrition

## Abstract

**Background:**

Phenylcapsaicin (PC) may enhance high-intensity exercise performance by reducing perceived exertion, increasing mechanical output, and limiting muscle damage, making it potentially beneficial for CrossFit^®^ (CF) athletes.

**Objective:**

To examine the acute effects of PC supplementation on performance, recovery, and metabolic responses during a CF session.

**Methods:**

This study had a randomized, triple-blind, placebo-controlled crossover design. Fifty CF-trained athletes (50% women) ingested either 2.5 mg of PC or a placebo (PLA) 45 minutes before a standardized CF session, including a warm-up, weightlifting block, and WOD. Delayed-onset muscle soreness (DOMS) was assessed 24- and 48-hours post-session. Countermovement jump (CMJ) was evaluated pre- and post-session, while a deep squat at 70% 1RM was performed post-session. Throughout the session, heart rate, capillary lactate, rating of perceived exertion (RPE), and perceived recovery status (PRS) were monitored.

**Results:**

Compared to PLA, PC improved squat performance at 70% 1RM in both load and repetitions (*P* ≤ 0.035), attenuated the decline in CMJ (*P* < 0.001), and maintained weightlifting performance over time (*P* interaction = 0.011), with significantly higher load in round 9 (*P* = 0.030). No differences were observed during the WOD (*P* interaction ≥ 0.826). DOMS was significantly lower in the PC group at both 24 h and 48 h (*P* = 0.030), while no group differences were found for lactate, RPE, PRS, or heart rate (*P* interaction ≥ 0.340). Analysis stratified by sex showed that PC reduced CMJ loss in men (*P* = 0.043) and increased squat load in women (*P* = 0.021).

**Conclusion:**

In conclusion, acute PC supplementation enhances performance and recovery in CF athletes.

## 1. Introduction

Concurrent training has gained widespread popularity due to its benefits for cardiometabolic health and improvements in body composition [1]. Among the various modalities of concurrent training, CrossFit^®^ (CF) [2] has emerged as a prominent and rapidly expanding form of high-intensity training [3]. CF is structured around the Workout of the Day (WOD) and typically incorporates whole-body movements drawn from disciplines such as gymnastics, weightlifting, and/or endurance training [4]. These exercises are often combined in high-intensity workouts and are performed with rapid execution, frequent repetitions, and little or no recovery [3]. The diverse and varied nature of this sport, combined with the unpredictability of competitive events, which are often not disclosed beforehand, demands that athletes be prepared for unexpected physical challenges. As a result, CF athletes must develop multiple fitness capacities to a high level, such as maximal strength, endurance, power, speed, and cardiorespiratory capacity [5,6]. CF sessions are characterised by elevated heart rate (HR), oxygen consumption, total energy expenditure, ratings of perceived exertion, and lactate levels both during and immediately after exercise [7,8]. Likewise, due to the typically intermittent and intense nature of CF sessions, energy production during exercise is primarily reliant on the phosphagen and glycolytic pathways [9].

Given the high physiological demands of CF, there is a growing interest in optimising energy availability and performance. In this context, the supplement market is currently innovating with new nutraceuticals and compounds that can enhance performance. One such compound is capsaicin (8-methyl-*N*-vanillyl-trans-6-nonenamide), the most abundant capsaicinoid in spicy chilli peppers, characterised by its vanilloid structure [10]. The physiological effects of capsaicin are attributed to its interaction with the transient receptor potential vanilloid 1 (TRPV1) [11]. TRPV1 is particularly abundant in type III and IV afferent nerve fibres, which are associated with peripheral and central fatigue during high-intensity exercise [12]. Concretely, activating TRPV1 in these nerve fibres has been proposed as a target for improving physical performance through capsaicin supplementation [13,14]. In skeletal muscle, capsaicin may enhance muscle contraction by increasing perceived heat-induced analgesia and promoting calcium release from the sarcoplasmic reticulum, both linked to motor unit function [11]. Indeed, capsaicin may improve sports performance by reducing perceived exertion, increasing mechanical performance (e.g., total volume load), and decreasing muscle damage [14,15]. However, a common side effect of capsaicin supplementation is gastrointestinal discomfort, regardless of the method of administration [16]. Recently, a new synthetic analogue called phenylcapsaicin (PC) has emerged as a potential alternative to traditional oral purified capsaicin supplementation [17]. PC is usually marketed as a microencapsulation composed of 98% PC and 1-1.5% cellulose and lipid excipients as primary carriers [17]. This microencapsulation process may result in lower pungency, reduced irritation of the digestive mucosa, and potentially improved bioavailability compared to orally purified capsaicin [17,18]. As the ergogenic dose of purified capsaicin is around 12 mg [13], PC may exert positive ergogenic effects at lower doses (>2.5 mg) due to this microencapsulation, which is rapidly distributed to tissues such as the small intestine, stomach, and liver within 30 minutes of ingestion [17]. Previous studies have demonstrated that PC supplementation improves strength and aerobic performance, reduces the rating of perceived exertion, and mitigates muscle damage in trained men [14,15,19]. However, to date, no study has investigated the acute effects of PC supplementation on CF performance. Given the close relationship between the metabolic and physiological responses of CF, where perceived exertion, lactate production, and muscle damage are elevated, it could be expected that PC supplementation, whose mechanisms of action target these parameters, may exert a favourable effect on performance, recovery, and metabolic outcomes in CF.

Therefore, this randomised, triple-blind, placebo-controlled crossover trial aimed to investigate the effect of acute PC supplementation on performance, recovery, and metabolic parameters during a CF session in male and female CF athletes.

## 2. Methods

### 2.1. Experimental design

**Figure 1.**
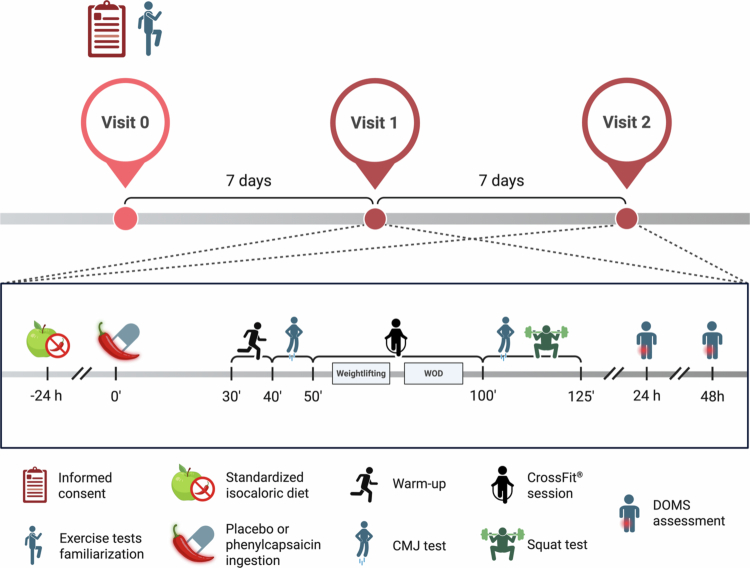
Overview of the study design. Abbreviations: CMJ, countermovement jump; DOMS, delayed-onset muscle soreness; WOD, Workout of the Day.

This study had a randomised, triple-blind, counterbalanced, placebo-controlled, crossover design, which included three sessions (one preliminary session and two experimental sessions; Figure 1) (ClinicalTrials.gov: No. NCT06784271) and followed the CONSORT guidelines for crossover trials (Table S1). Participants, research staff, and statistical staff were blinded to the intervention conditions prior to and throughout data collection, curation, and analysis. Each participant completed two experimental sessions separated by a one-week washout period, with each session differing solely in the supplement provided [placebo (PLA) or PC]. The PLA and PC were anonymously coded and blinded by an external researcher not involved in the current study. All participants provided written informed consent after being thoroughly informed about the study procedures. The study was approved by the Human Research Ethics Committee of the “Investigación con Medicamentos (CEIm) del Complejo Universitario de Canarias” (no. CHUC_2024_80) and was conducted in accordance with the latest revision of the Declaration of Helsinki. All experimental procedures took place at CrossFit Omicron (Tenerife, Spain) between November and January 2024−2025. There were no changes after the trial commencement.

### 2.2. Participants

Twenty-five male and twenty-five female CF-trained adults participated in the present study. We recruited the participants from October to December 2024 through social media and poster advertisements in Tenerife, Spain. Inclusion criteria included: (i) age between 18-35 years; (ii) body mass index between 18.5-30 kg/m^2^; (iii) having a minimum of 3 years of experience in CF with at least 6 hours of training per week for the last 3 months; and (iv) residency of Tenerife. Exclusion criteria encompassed: (i) the presence of major chronic diseases, pregnancy, breastfeeding, or hypersensitivity to capsaicin; (ii) any acute or chronic condition that may be affected by the use of nutritional supplements or exercise, especially pungent components; (iii) body weight fluctuations exceeding 4 kg in the last three months before the study onset; or (iv) any circumstance that may compromise the validity of the study findings or the safety of participants.

### 2.3. Supplementation procedures

Based on each experimental condition, participants ingested four capsules with water 45 minutes before exercise. The capsules contained either a total of 2.5 mg of PC (Axitite, Malmö, Sweden) or a placebo (PLA), consisting of 600 mg of maltodextrin, microcrystalline cellulose, and magnesium stearate as excipients. Each condition was packaged and labelled with a randomised numeric code provided by the manufacturer (Life Pro Nutrition Industries, Madrid, Spain). The order of administration (PLA or PC) was determined using simple randomisation prior to data collection. Specifically, a researcher not involved in participant testing generated the allocation sequence with the random sample function in MS Excel®. The PLA and PC capsules were identical in flavour and visual appearance.

### 2.4. Preliminary session (session 1)

We held a prior meeting with the participants to read and sign the informed consent, as well as to confirm the eligibility criteria through questionnaires. Subsequently, body mass, fat mass, and muscle mass (Tanita Innerscan V, model BC-545N, Amsterdam, The Netherlands) and height (Seca model 799, Electronic Column Scale, Hamburg, Germany) were evaluated following the manufacturer's recommendations. Participants were asked to report their one-repetition maximum (1RM) for the deadlift and back squat, as well as their total years of training experience. They were also familiarised with the countermovement jump (CMJ) measured using the Chronojump app (Chronojump Boscosystem, Cataluña, Spain) [20] and with the deep squat test performed at 70% of their 1RM calculated using the two-point method [21], and measured with a Vitruve linear encoder (Vitruve, Sevilla, Spain) [22]. Additionally, the participants were instructed to follow a standardised isocaloric diet (1.8 g protein/kg body mass; 7 g carbohydrates/kg body mass; and fats to complete the estimated energy requirements) that was replicated during the 24 h preceding each experimental session. The energy requirements were estimated using the World Health Organisation equation (WHO) (1985) [23], multiplying the obtained result by a physical activity factor of 1.7.

### 2.5. Experimental sessions (sessions 2-3)

Before starting each experimental session, participants were verbally asked about their compliance with the following conditions: a) abstaining from vigorous physical activity within the 48 hours preceding the session, b) maintain regular sleep patterns, c) avoiding stimulants, such as caffeine or similar substances, at least 12 hours before the session, d) refraining from alcohol or drugs use within 24 hours before the session, e) adhering to the individualised diet during the 24 hours before the session and f) avoiding consumption of pungent food (e.g. chilli, mustard, pepper) at least 12 hours before the session. Adherence to these conditions was verified verbally at the beginning of each session, when participants were asked to confirm compliance with the instructions.

Participants attended at the same time for each session (from 10 to 13 am) to minimise potential circadian rhythm disruptions. Each session started with a specific warm-up protocol for the CMJ, which consisted of three rounds of 30 seconds of wall sit, 20 lunges, and 10 jumping squats, followed by three CMJ attempts, from which the average was calculated [24]. Afterward, participants completed a specific warm-up for the squat clean and jerk, consisting of muscle cleans, slow cleans, front squats, squat cleans, shoulder presses, and push jerks, performed in three rounds of five repetitions each using a 20 kg barbell.

The session included a weightlifting protocol, where participants performed one round every 1:45 minutes for nine total rounds. Each round involved 10 bar-facing burpees followed by one squat clean and jerk, starting at 50% of the participant’s self-reported 1RM and progressing to a maximum load.

After a seven-minute rest period, participants proceeded to complete the Open 21.3 WOD, which had a 15-minute time cap. This WOD used a barbell load of 43 kg for men and 30 kg for women, and included the following sequence: 15 front squats, 30 toes to bar, 15 thrusters, one minute of rest, 15 front squats, 30 chest-to-bar pull-ups, 15 thrusters, one minute of rest, 15 front squats, 30 bar muscle-ups, and 15 thrusters. For some female athletes, the gymnastic movements were scaled to toes-to-bar, pull-ups, and chest-to-bar in both sessions. A judge recorded all repetitions of each participant throughout the entire session. Athlete motivation and psychological readiness were not formally assessed. In addition, while verbal encouragement from judges was allowed during the sessions, the amount and content of feedback were not standardised or quantified.

### 2.6. Performance assessment

The CMJ test was performed both before and immediately after the CF session. Flight time, jump height, power, and jump speed were measured with the Chronojump app [24].

Furthermore, the weight lifted, number of repetitions, mean propulsive velocity, mean velocity, and faster repetition during the 70% RM squat test were measured using a Vitruve linear encoder [22]. The test was conducted on both experimental days after the CF session. Participants started with back squats at 20 kg, and the load was gradually increased by 5 to 20 kg increments, with 3-minute rest periods between sets. Sets were performed until the mean barbell velocity reached 0.5 and 0.6 m/s. Subsequently, the 1RM was estimated using the two-point method [21], and participants performed back squats to failure at 70% of the calculated 1RM.

### 2.7. CrossFit® performance

CF performance was measured in the weightlifting protocol, registering the load lifted in each round, the load lifted in the last round (nº 9), and the number of rounds performed without failing the lift. Failure was defined as the inability to complete the lift with correct technique through the full range of motion or the inability to lift the assigned load despite verbal encouragement. Likewise, in the WOD, performance was registered by the number of front squats, thrusters, or toes-to-bar/chest-to-bar/muscle-up performed in each round.

### 2.8. Delayed-onset muscle soreness assessment

Delayed-onset muscle soreness (DOMS) was assessed using a previously validated visual analogue scale [25]. Participants were instructed to mark from 0 to 10 cm on a horizontal line, where 0 indicated “no pain”, and 10 indicated “unbearably painful”. This scale was administered 24 and 48 hours after the CF sessions.

### 2.9. Ratings of perceived exertion and perceived recovery status

Ratings of perceived exertion (RPE) were assessed after each block (i.e., warm-up, weightlifting, WOD, and back squat) using the validated Borg scale (0−10) [26]. Similarly, the perceived recovery status (PRS) was evaluated after each block (i.e., warm-up, weightlifting, WOD, and back squat) using a previously validated scale (10−0) [27].

### 2.10. Heart rate and lactate assessments

HR was measured during each block (weightlifting and WOD) using a Garmin HRM-Dual™ (Garmin Ltd., Kansas, United States). The data were averaged using Heart Rate Monitor 1.2.5 (BM innovations GmbH, Hörgertshausen, Germany), and the mean value for each minute was calculated during the weightlifting and the WOD. Capillary lactate was measured with a finger prick after the weightlifting and WOD blocks using the Lactate Pro 2 (Arkray, Kyoto, Japan).

### 2.11. Statistical analyses

The sample size was estimated using G*Power software (version 3.1.9.7, University of Düsseldorf, Germany). A total of 20 participants were needed to determine statistically significant differences between conditions (PLA vs. PC) in CMJ (~5%) with a statistical power of 80% at *α* = 0.05 [28]. To account for planned analyses stratified by sex, and considering our previous experience with similar exercise protocols in which participant losses due to scheduling conflicts, acute injuries, or non-adherence were not negligible, we conservatively assumed a dropout rate of 25%. Therefore, we recruited a total of 50 participants (50% women) to ensure adequate statistical power.

Descriptive data are expressed as mean and standard deviation (SD). Repeated-measures linear mixed models were conducted to compare the effects of PLA and PC on the dependent outcomes. Model-based estimations were performed with an intention-to-treat approach using the restricted maximum-likelihood method, the model assuming that missing values were missing at random. Benjamini-Hochberg post hoc adjustments for multiple comparisons were used to examine differences between groups. In addition, we conducted sensitivity and post hoc analyses by performing the same repeated-measures linear mixed models but stratifying the analysis by sex (i.e., separately for men and women). Model residuals were visually inspected through Q–Q plots and histograms, and their distribution did not deviate substantially from normality. Homoscedasticity was also assessed by plotting residuals against fitted values. The level of statistical significance was set at *P* < 0.05.

All analyses were conducted using R software, version 4.1.2 (https://cran.r-project.org/, The R Project for Statistical Computing, Vienna, Austria); linear mixed-effects models were performed using the lme4 package for R software (version v1. 1–26).

## 3. Results

A total of 50 participants, 25 men and 25 women, completed the study (Table 1). The descriptive characteristics of the participants can be found in Table 1. There were no excluded participants.

**Table 1.** Descriptive characteristics of the study participants.

	All (*n* = 50)		Women (*n* = 25)		Men (*n* = 25)	
	Mean	SD	Mean	SD	Mean	SD
Age (years)	30.2	3.7	29.5	4.1	30.9	3.1
Body mass (kg)	75.0	11.5	66.3	7.1	83.6	7.8
Height (cm)	170.7	9.0	165.2	7.2	176.3	6.8
Fat mass (%)	17.9	4.8	21.1	3.7	14.6	3.3
Muscle mass (kg)	59.5	11.3	49.7	5.4	69.2	5.8
Training experience (age)	4.9	1.9	4.3	1.3	5.4	2.3
1RM Back squat (kg)	132.0	33.1	105.4	16.6	158.6	22.2
1RM deadlift (kg)	155.2	37.9	123.2	16.0	187.2	23.2

Data presented as mean and standard deviation (SD). Abbreviations: 1RM, one repetition maximum; BMI, body mass index.

**Figure 2.**
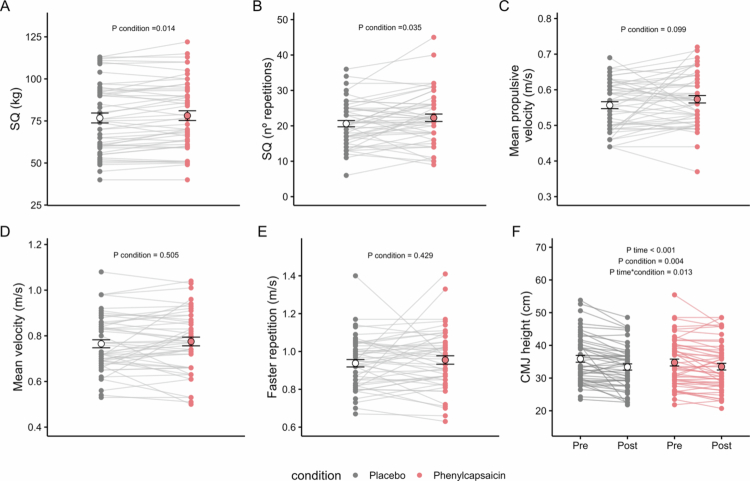
Effects of acute phenylcapsaicin supplementation on the 70% RM squat test and countermovement jump test. Weight (A), number of repetitions (B), mean propulsive velocity (C), mean velocity (D), faster repetition (E) of the 70% RM squat test, and CMJ height (F) after placebo (grey), or phenylcapsaicin (red) supplementation. Data are depicted as mean ± SD. *P* values obtained from linear mixed repeated-measures analyses. Abbreviations: CMJ, countermovement jump; SQ, squat.

A higher load and number of repetitions were observed in the squat at 70% of 1RM after PC supplementation compared to PLA (78.5 kg and 22.3 repetitions; and 76.4 kg and 20.7 repetitions, respectively; *P* condition ≤ 0.035; Figure 2A and B). However, no significant differences were observed in mean propulsive velocity, mean velocity, or fastest repetition between PC and PLA (all *P* condition ≥ 0.099; Figure 2C−E). Additionally, the CF session significantly decreased CMJ height (*P* time < 0.001), but compared to PLA, PC supplementation reduced the decrease in CMJ height (*P* time*condition = 0.013; PLA = −2.25 ± 0.47 cm, PC = −1.26 ± 0.46 cm, pairwise comparison *P* = 0.054; Figure 2F). There were no differences in CMJ power, flight time, or velocity (all *P* time*condition ≥ 0.202, Figure S1). When we repeated the analysis separately for men (Figure S2) and women (Figure S3), we observed that PC supplementation reduced the decrease in CMJ height induced by the CF session in men (*P* time*condition = 0.043; Figure S2F), and increased the load in the squat at 70% of 1RM in women (*P* condition = 0.021; Figure S3A) compared to PLA.

**Figure 3.**
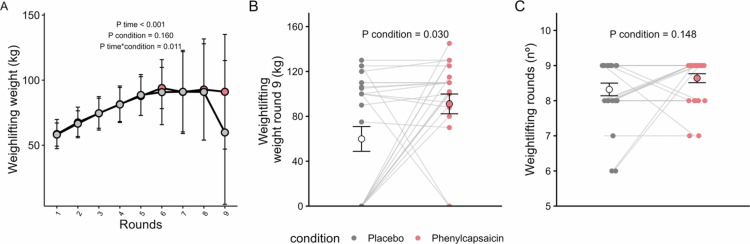
Effects of acute phenylcapsaicin supplementation on weightlifting performance. Weight per round (A), weight in round 9 (B), and number of rounds (C) after placebo (grey), or phenylcapsaicin (red) supplementation. Data are depicted as mean ± SD. *P* values obtained from linear mixed repeated-measures analyses.

Furthermore, PC supplementation maintained weightlifting performance over weightlifting rounds compared to PLA (*P* time*condition = 0.011; Figure 3A), with a significantly higher weight lifted in round 9 (73.1 kg and 55.5 kg, respectively; *P* condition = 0.030; Figure 3B). However, no significant differences were observed in the number of rounds performed in the weightlifting block (*P* condition = 0.403, Figure 3C). These results persisted when we repeated the analysis separately in men (Figure S4), but not in women, where we observed no significant differences in weightlifting performance between conditions (Figure S5).

**Figure 4.**
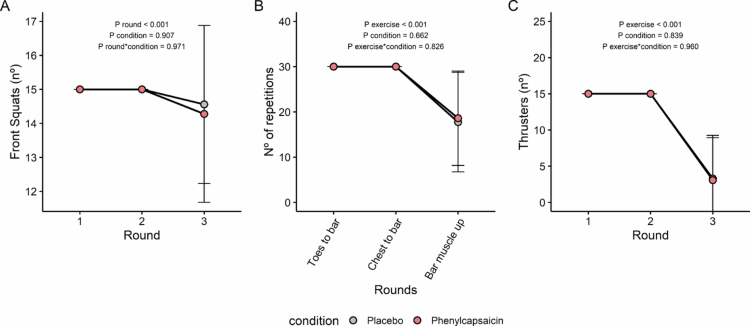
Effects of acute phenylcapsaicin supplementation on WOD performance. Number of front squats (A), number of toes to bar, chest to bar or bar muscle up (B), and number of thrusters (C) after placebo (grey), or phenylcapsaicin (red) supplementation. Data are depicted as mean ± SD. *P* values obtained from linear mixed repeated-measures analyses.

In turn, no differences between PC and PLA were observed during the WOD (i.e., nº of front squats, toes-to-bar/chest-to-bar/bar-muscle-up or thrusters; all *P* time*condition ≥ 0.826, Figure 4). These results persisted when we repeated the analysis separately in men (Figure S6) and in women (Figure S7).

**Figure 5:**
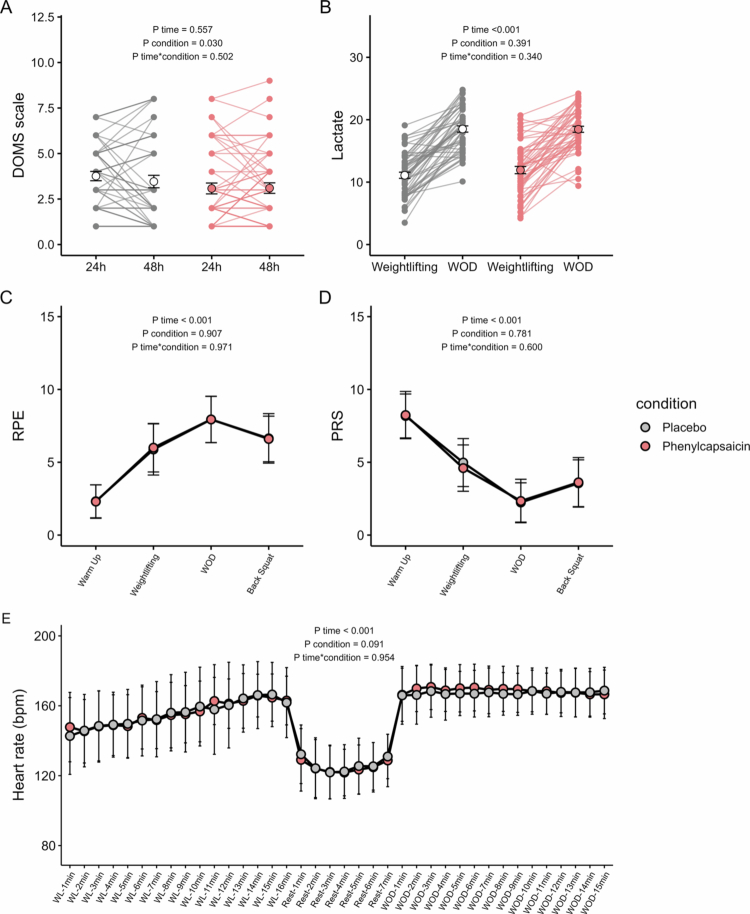
Effects of acute phenylcapsaicin supplementation on subjective and metabolic parameters. DOMS scale (A), lactate (B), RPE (C), RPS (D), and heart rate (E) after placebo (grey), or phenylcapsaicin (red) supplementation. Data are depicted as mean ± SD. *P* values obtained from linear mixed repeated-measures analyses. Abbreviations: bpm, beats per minute; RPE, rate of perceived effort; PRS, perceived recovery status.

In addition, we observed a lower DOMS scale score after PC supplementation compared to PLA at 24 h and 48 h after the CF session (24 h: 3.08 and 3.76 PC and PLA, respectively, *P* = 0.045; 48 h: 3.10 and 3.46 PC and PLA, respectively, *P* = 0.286; *P* condition = 0.030; Figure 5A). However, no time*condition interaction was found in the DOMS scale (Figure 5A). Lactate levels, RPE, RPS, and HR increased over the CF session (all *P* time < 0.001), but we observed no significant differences between PC and PLA supplementation (all *P* time*condition ≥ 0.340, Figure 5B−E).

Lastly, six participants (12%) reported heartburn or acid reflux after PC supplementation. No adverse event was recorded after PLA supplementation.

## 4. Discussion

This randomised, triple-blind, placebo-controlled, crossover trial found that acute PC supplementation could improve exercise performance and recovery after a CF session compared to PLA. Additionally, PC supplementation maintained weightlifting performance during the CF session and reduced fatigue and DOMS after the session. Lastly, while some performance and recovery variables showed sex-specific responses to PC supplementation, the overall improvements in exercise performance and recovery were observed consistently across both sexes. Collectively, these results provide evidence supporting acute PC supplementation as a new ergogenic supplement to optimise exercise performance and expedite recovery.

The observed improvements in squat load and repetitions, and weightlifting performance, align with previous findings in resistance training, supporting the efficacy of PC supplementation in enhancing strength-related outcomes. In this sense, the supplementation of 2.5 mg of PC in twenty-five trained athletes increased the total number of squats repetitions performed to failure in a 4 × 70% 1RM protocol [15]. Similarly, eleven trained individuals completed an average of 4.4 more repetitions (21% increase) in the squat test at 70% 1RM [29], consistent with our findings. Moreover, young training men ingesting 12 mg of capsaicin increased the number of repetitions performed throughout four sets of back squats to muscle failure at 70% 1RM compared to PLA [13]. PC supplementation has also been shown to enhance CMJ height in twenty-five trained athletes compared to PLA [19]. The improvements on strength-related outcomes may be explained by the activation of TRPV1 receptor by PC, with TRPV1 activation leading to increased calcium release, enhancing muscle contraction and contributing to an analgesic effect, which may confer greater force production and tolerance to strenuous exercise [11,30]. Finally, TRPV1 activation may also reduce the muscular cost of adenosine triphosphate, which could be associated with reduced fatigue [31,32]. These same mechanisms may have contributed to improved weightlifting performance, particularly during the 9th round of lifting after PC supplementation. Reaching the final round with reduced fatigue and comparable RPE to the PLA conditions may have enabled athletes to lift heavier loads.

On the contrary, we did not observe improvements in WOD performance. These findings contrast with previous studies involving high-intensity exercise. Supplementation with 12 mg of capsaicin increased time to exhaustion during high-intensity interval exercise (HIIE) in a 13% [33]. Similarly, in another study, eleven trained men completed a 5-km interval run (1-minute bouts at maximal aerobic power interspersed with 1-minute passive recovery), and capsaicin supplementation led to a 20.8% increase in total volume compared to PLA [29]. Additionally, in a 1500-metre run test involving physically active individuals, capsaicin supplementation significantly improved time to completion [34]. The discrepancy between our findings and those of previous studies may be explained by several factors: i) the capsaicin employed (capsaicin instead of PC); ii) the complexity of the WOD, which included technical elements such as bar muscle-ups or chest-to-bar pull-ups that may have affected performance; iii) the higher physical demand in the WOD compared to previously studied protocols, as observed in the herein recorded RPE values which are representative of CF [8]; iv) and the gastrointestinal discomfort—specifically heartburn—reported by six participants during the WOD, which may have negatively influenced performance. Lastly, the familiarisation effect should be considered [35], as performance improvements in repeated tasks are often due not to physiological adaptations, but to increased task familiarity, reduced anxiety, improved pacing, or better technical execution [36].

Despite lifting more weight and repetitions in the squat test and weightlifting, we observed lower DOMS scores both 24- and 48-hours post-exercise after PC supplementation compared to PLA. These findings are consistent with a previous study in which 12 futsal players were given either 12 mg of capsaicin or PLA and performed 200 jumps while wearing a weighted vest equivalent to 10% of their body weight. PC supplementation led to reduced DOMS scores at 12-, 24-, and 48-hours post-exercise [37]. This effect may be partially mediated because Capsaicin supplementation may help reduce exercise-induced inflammation by blunting the rise of interleukin-1β (IL-1β) following exercise [35].

Conversely, we found no differences in RPE after PC supplementation, aligning with previous studies involving repeated sprints interspersed with recovery in trained subjects [16,33]. However, other high-intensity training protocols (i.e., 5-km intermittent run and 1,500-metre run) have reported improvements in recreationally resistance-trained men and physically active men [29,34]. Similarly, no differences were observed in RPS or HR, which contrasts with one study reporting a decrease in HR following supplementation in a 5-km test (1:1 effort and pause ratio) [29]. Additionally, we found no changes in lactate levels after PC supplementation, a finding replicated in two other studies involving in 1,500m run and 15 seconds at 120% of sVO_2Peak_, interspersed by 15 seconds of passive recovery in physically active men [33,34]. These mixed findings may be attributed to the inherently high intensity of the exercise, which likely elevated perceived exertion, subjective recovery, HR, or lactate across all conditions regardless of the supplementation protocol [38].

In addition, when analysing the results separated by men and women, we observed some sex-dependent benefits after PC supplementation. Specifically, we showed that men improved weightlifting performance over weightlifting rounds and reduced the decrease in CMJ height, whereas women improved the load lifted in the 70% squat and DOMS scale. This sex-dependent disparity after PC supplementation may be attributed to women’s tendency to exhibit higher TRPV1-mediated pain sensitivity due to oestrogen, whereas men might experience more effective inhibition of this receptor via testosterone [39]. However, future studies should analyse sex differences in exercise performance, recovery, and metabolic parameters after PC supplementation.

### 4.1. Practical implications

From an applied perspective, PC supplementation may enhance performance by increasing the number of repetitions in exercises such as the squat and attenuating fatigue. This improvement can translate into greater work capacity, as it enables a higher total training volume and facilitates recovery between sessions—an aspect of particular relevance in high-demand disciplines such as CF, which are characterised by substantial training loads. Moreover, this potential benefit may hold competitive significance, since performance differences between first and second place are often determined by only one or two additional repetitions. However, a possible limitation in the practical implementation of PC supplementation is the occurrence of gastrointestinal discomfort; therefore, it is advisable to conduct prior testing to determine each athlete’s individual tolerance.

### 4.2. Strengths and limitations

The main strength of the present study was the randomised, triple-blind, placebo-controlled, crossover design, with diet standardisation 24 hours before the intervention. In addition, our study participants were trained male and female CF athletes. This study is not without limitations, such as the absence of biochemical indicators like creatine kinase or inflammatory cytokines to evaluate the EIMD. Therefore, additional research is required to address these limitations and assess the role of PC ingestion in CF athletes.

## 5. Conclusion

In conclusion, acute PC supplementation maintains weightlifting performance during a CF session, reduces fatigue and DOMS after the session, increases squat load in women, and reduces CMJ height loss in men without modifying metabolic parameters during exercise. Together, these findings introduce novel evidence positioning PC supplementation as a promising, acute ergogenic aid capable of enhancing performance and accelerating recovery. However, future studies should evaluate different doses of PC supplementation and types of training sessions, varying in exercises and duration, and conducted in elite CF athletes.

## Supplementary Material

Supplementary Material SUPPLEMENTARY_MATERIAL_R1clean_-_Copy.docx

## Data Availability

The data that support the findings of this study are available from the corresponding author, LJ-F, upon reasonable request.
