# Supplementary material for: Acute phenylcapsaicin supplementation improves CrossFit® performance: a randomized, triple-blind, placebo-controlled crossover trial
Source: J Int Soc Sports Nutr. 2026 Jan 14;23(1):2615274. doi: 10.1080/15502783.2026.2615274 (PMC12810409; doi:10.1080/15502783.2026.2615274)
Supplement: Supplementary Material — SUPPLEMENTARY_MATERIAL_R1clean_-_Copy.docx [file RSSN_A_2615274_SM0453.docx]

SUPPLEMENTARY MATERIAL

Table S1. CONSORT checklist for crossover trials.

| Section/topic | Item No | Description | Page No* |
| --- | --- | --- | --- |
| Title | 1a | Identification as a randomised crossover trial in the title | 1 |
| Abstract | 1b | Specify a crossover design and report all information outlined in table 2 | 2 |
| Introduction: |  |  |  |
| Background | 2a | Scientific background and explanation of rationale | 3-4 |
| Objectives | 2b | Specific objectives or hypotheses | 5 |
| Methods: |  |  | 6 |
| Trial design | 3a | Rationale for a crossover design. Description of the design features including allocation ratio, especially the number and duration of periods, duration of washout period, and consideration of carry over effect | 6 |
| Change from protocol | 3b | Important changes to methods after trial commencement (such as eligibility criteria), with reasons | 6 |
| Participants | 4a | Eligibility criteria for participants | 6-7 |
| Settings and location | 4b | Settings and locations where the data were collected | 6 |
| Interventions | 5 | The interventions with sufficient details to allow replication, including how and when they were actually administered | 6 |
| Outcomes | 6a | Completely defined prespecified primary and secondary outcome measures, including how and when they were assessed | 5 |
| Changes to outcomes | 6b | Any changes to trial outcomes after the trial commenced, with reasons | 6 |
| Sample size | 7a | How sample size was determined, accounting for within participant variability | 11 |
| Interim analyses and stopping guidelines | 7b | When applicable, explanation of any interim analyses and stopping guidelines | n/a |
| Randomisation: |  |  |  |
| Sequence generation | 8a | Method used to generate the random allocation sequence | 7 |
| Sequence generation | 8b | Type of randomisation; details of any restriction (such as blocking and block size) | 7 |
| Allocation concealment mechanism | 9 | Mechanism used to implement the random allocation sequence§ (such as sequentially numbered containers), describing any steps taken to conceal the sequence until interventions were assigned | 7 |
| Implementation | 10 | Who generated the random allocation sequence,§ who enrolled participants, and who assigned participants to the sequence of interventions | 7 |
| Blinding | 11a | If done, who was blinded after assignment to interventions (for example, participants, care providers, those assessing outcomes) and how | 7 |
| Similarity of interventions | 11b | If relevant, description of the similarity of interventions | n/a |
| Statistical methods | 12a | Statistical methods used to compare groups for primary and secondary outcomes which are appropriate for crossover design (that is, based on within participant comparison) | 7 |
| Additional analyses | 12b | Methods for additional analyses, such as subgroup analyses and adjusted analyses | 9-10 |
| Results |  |  |  |
| Participant flow (a diagram is strongly recommended) | 13a | The numbers of participants who were randomly assigned, received intended treatment, and were analysed for the primary outcome, separately for each sequence and period | 6 |
| Losses and exclusions | 13b | No of participants excluded at each stage, with reasons, separately for each sequence and period | 13 |
| Recruitment | 14a | Dates defining the periods of recruitment and follow-up | 6 |
| Trial end | 14b | Why the trial ended or was stopped | 6 |
| Baseline data | 15 | A table showing baseline demographic and clinical characteristics by sequence and period | 25 |
| Numbers analysed | 16 | Number of participants (denominator) included in each analysis and whether the analysis was by original assigned groups | 11 |
| Outcomes and estimation | 17a | or each primary and secondary outcome, results including estimated effect size and its precision (such as 95% confidence interval) should be based on within participant comparisons.¶ In addition, results for each intervention in each period are recommended | 11 |
| Binary outcomes | 17b | For binary outcomes, presentation of both absolute and relative effect sizes is recommended | 11 |
| Ancillary analyses | 18 | Results of any other analyses performed, including subgroup analyses and adjusted analyses, distinguishing prespecified from exploratory | 11 |
| Harms | 19 | Describe all important harms or untended effects in a way that accounts for the design (for specific guidance, see CONSORT for harms32) | 11 |
| Discussion: |  |  |  |
| Limitations | 20 | Trial limitations, addressing sources of potential bias, imprecision, and if relevant, multiplicity of analyses. Consider potential carry over effects | 18 |
| Generalisability | 21 | Generalisability (external validity, applicability) of the trial findings | 18 |
| Interpretation | 22 | Interpretation consistent with results, balancing benefits and harms, and considering other relevant evidence | 18 |
| Other information: |  |  |  |
| Registration | 23 | Registration number and name of trial registry | 6 |
| Protocol | 24 | Where the full trial protocol can be accessed, if available | 6 |
| Funding | 25 | Sources of funding and other support (such as supply of drugs), role of funders | 20 |


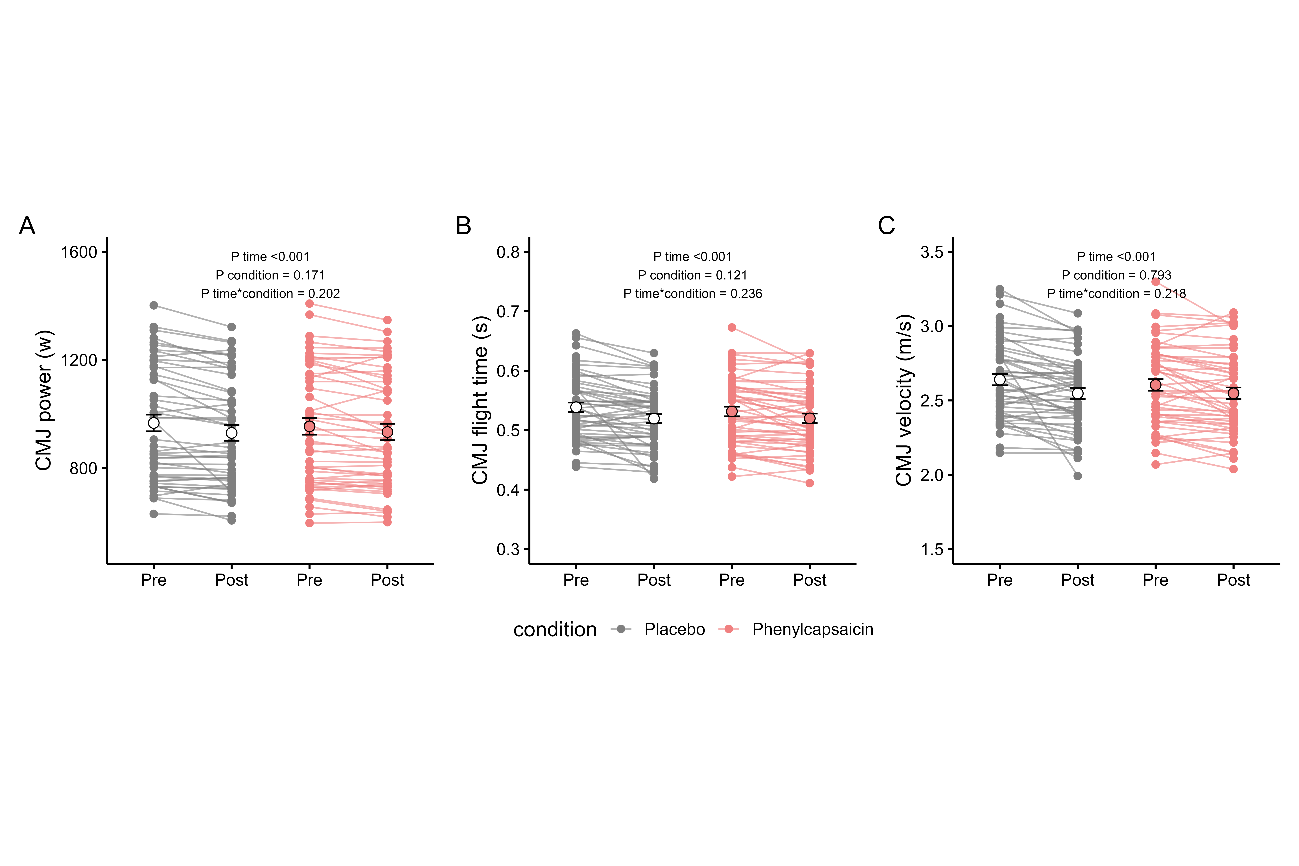


Figure S1. Effects of acute phenylcapsaicin supplementation on the countermovement jump test. Power (A), flight time (B), and velocity (C) after placebo (grey), or phenylcapsaicin (red) supplementation. Data are depicted as mean ± SD. P values obtained from linear mixed repeated-measures analyses. *Abbreviations*: CMJ, countermovement jump.


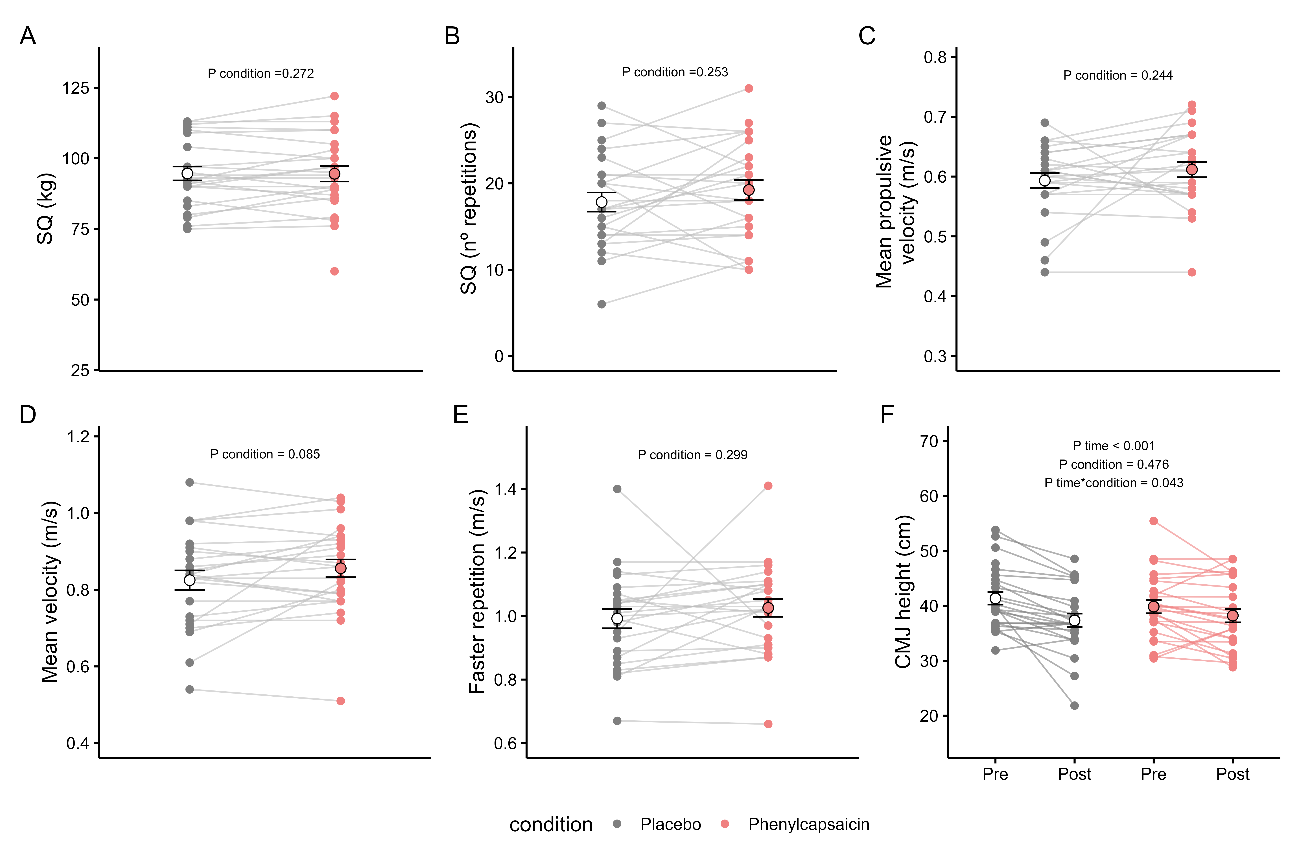


Figure S2. Effects of acute phenylcapsaicin supplementation on the 70% RM squat test and countermovement jump test in men. Weight (A), number of repetitions (B), mean propulsive velocity (C), mean velocity (D), faster repetition (E) of the 70% RM squat test, and CMJ height (F) after placebo (grey), or phenylcapsaicin (red) supplementation. Data are depicted as mean ± SD. P values obtained from linear mixed repeated-measures analyses. *Abbreviations*: CMJ, countermovement jump; SQ, squat.


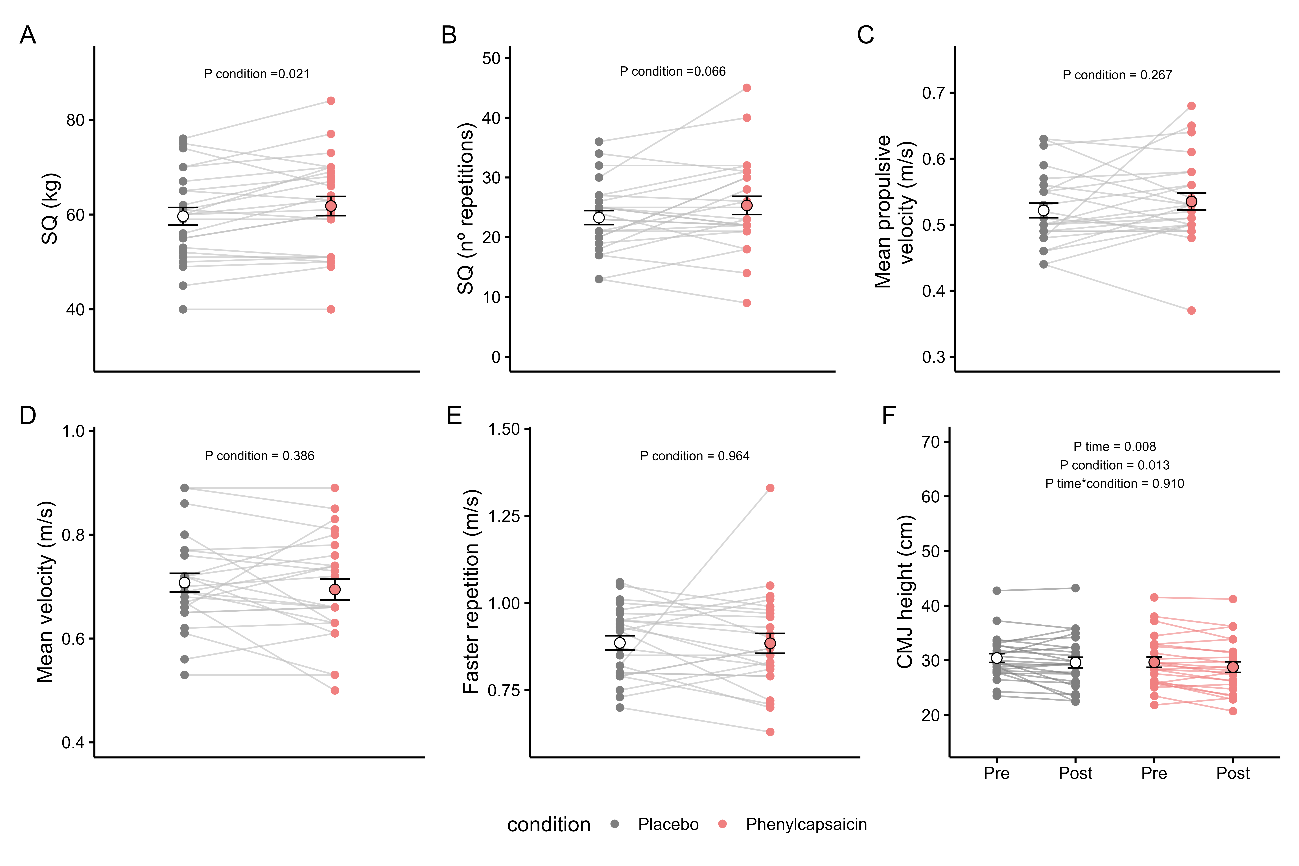


Figure S3. Effects of acute phenylcapsaicin supplementation on the 70% RM squat test and countermovement jump test in women. Weight (A), number of repetitions (B), mean propulsive velocity (C), mean velocity (D), faster repetition (E) of the 70% RM squat test, and CMJ height (F) after placebo (grey), or phenylcapsaicin (red) supplementation. Data are depicted as mean ± SD. P values obtained from linear mixed repeated-measures analyses. *Abbreviations*: CMJ, countermovement jump; SQ, squat.


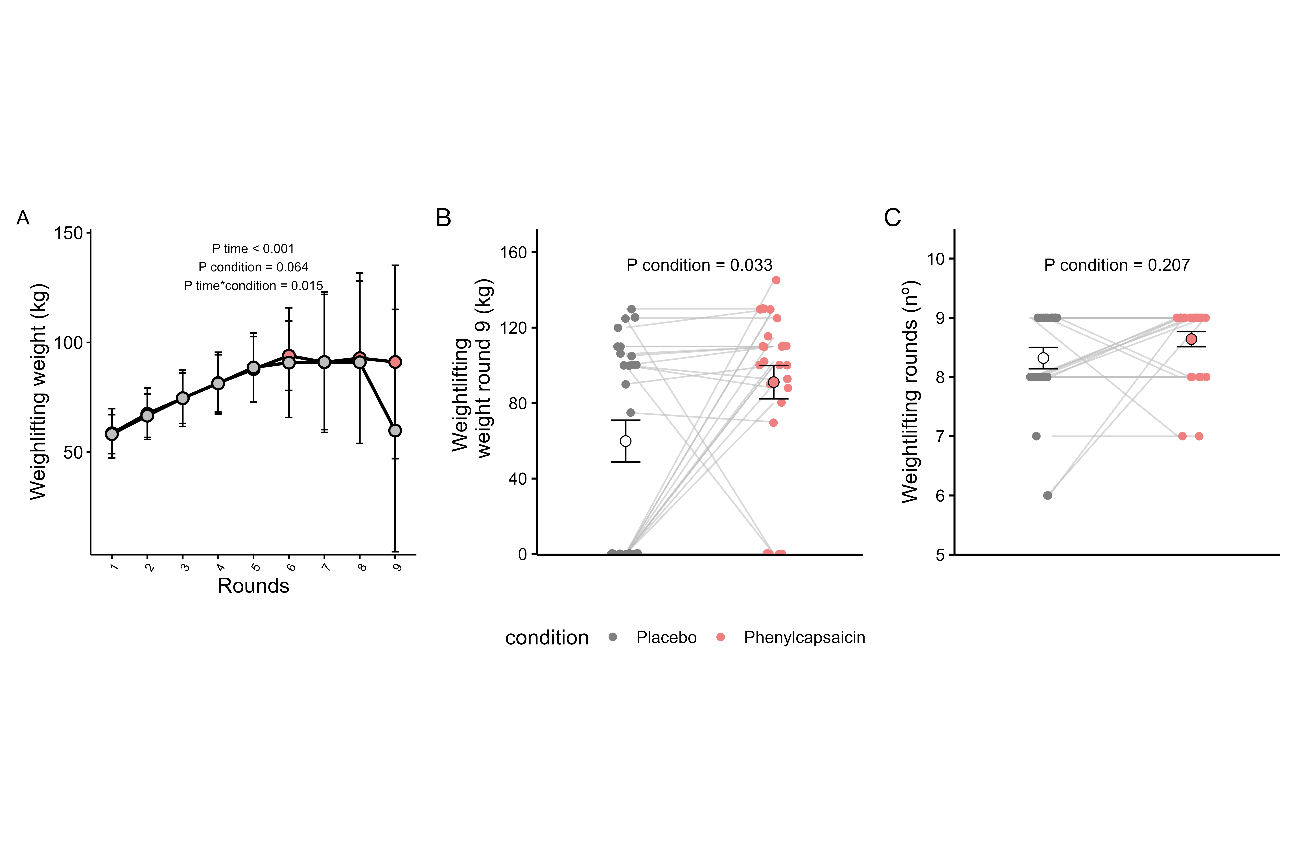


Figure S4. Effects of acute phenylcapsaicin supplementation on weightlifting performance in men. Weight per rounds (A), weight in the round 9 (B), and number of rounds (C) after placebo (grey), or phenylcapsaicin (red) supplementation. Data are depicted as mean ± SD. P values obtained from linear mixed repeated-measures analyses.


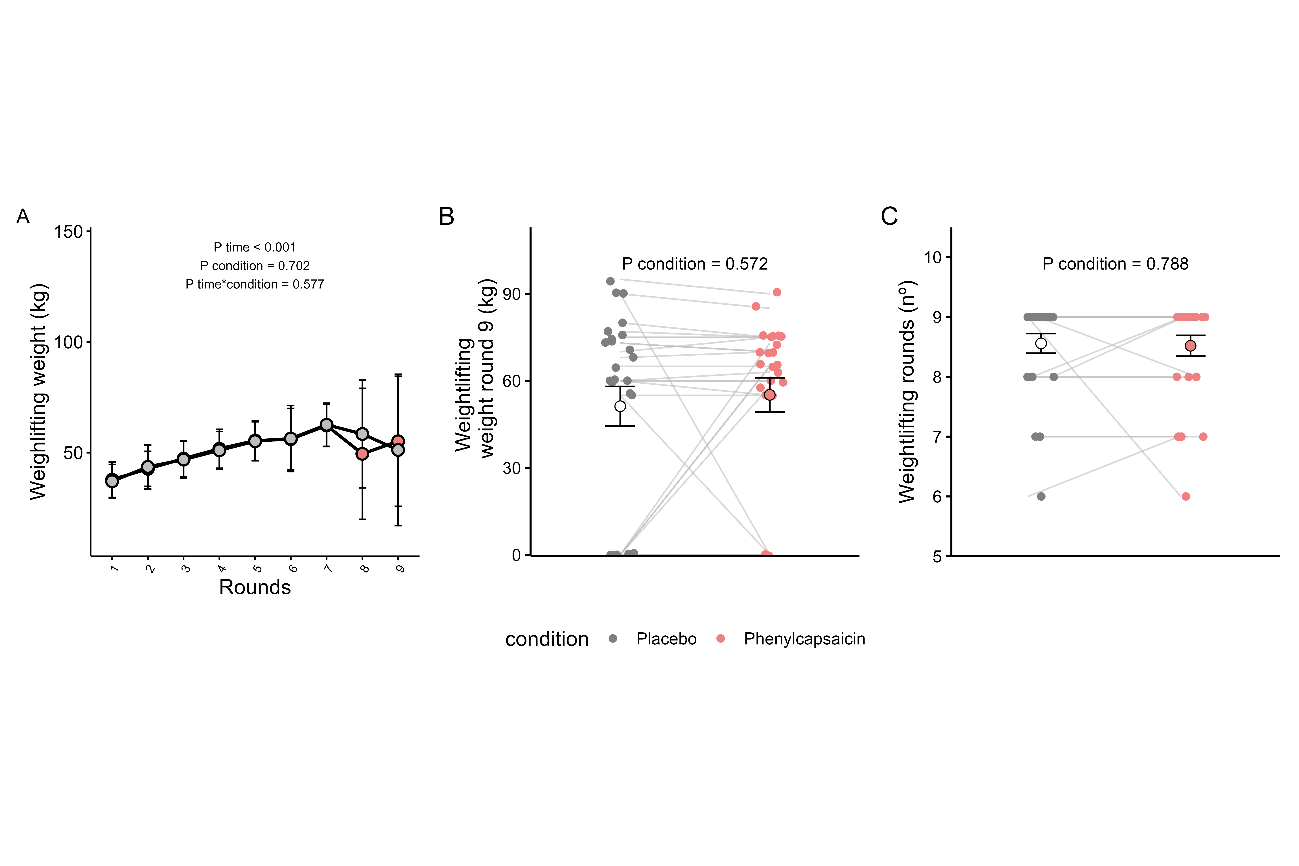


Figure S5. Effects of acute phenylcapsaicin supplementation on weightlifting performance in women. Weight per rounds (A), weight in the round 9 (B), and number of rounds (C) after placebo (grey), or phenylcapsaicin (red) supplementation. Data are depicted as mean ± SD. P values obtained from linear mixed repeated-measures analyses.


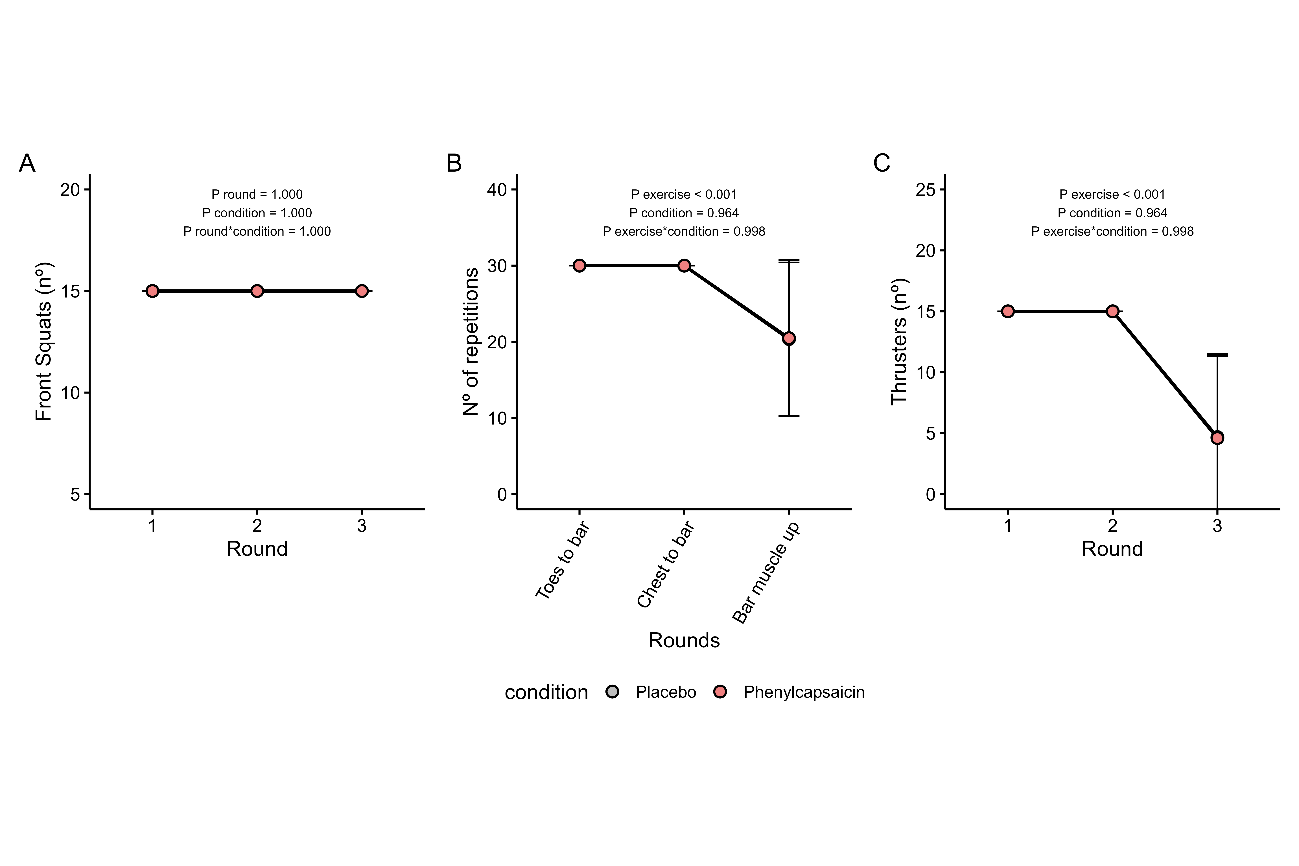


Figure S6. Effects of acute phenylcapsaicin supplementation on WOD performance in men. Number of front squats (A), number of toes to bar, chest to bar or bar muscle up (B), and number of thrusters (C) after placebo (grey), or phenylcapsaicin (red) supplementation. Data are depicted as mean ± SD. P values obtained from linear mixed repeated-measures analyses.


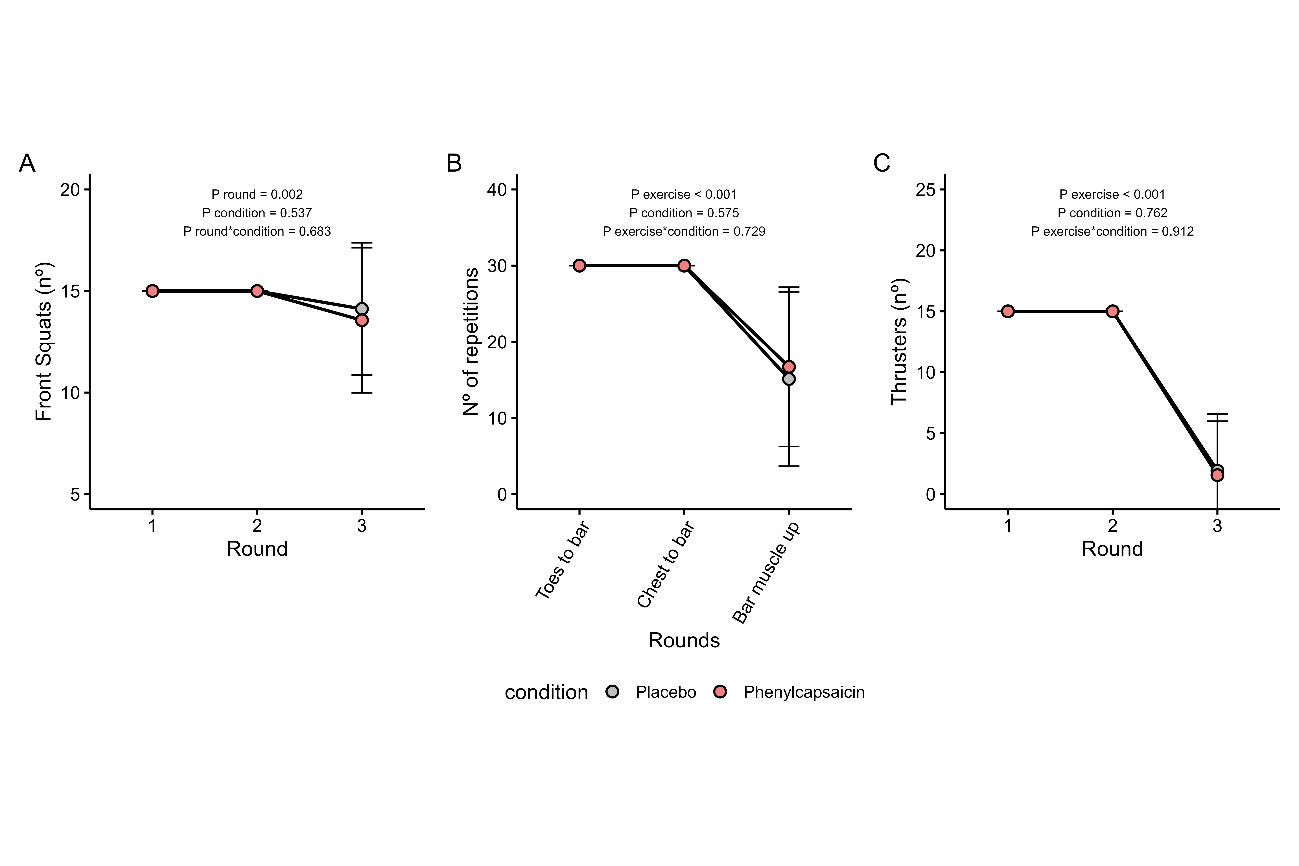


Figure S7. Effects of acute phenylcapsaicin supplementation on WOD performance in women. Number of front squats (A), number of toes to bar, chest to bar or bar muscle up (B), and number of thrusters (C) after placebo (grey), or phenylcapsaicin (red) supplementation. Data are depicted as mean ± SD. P values obtained from linear mixed repeated-measures analyses.


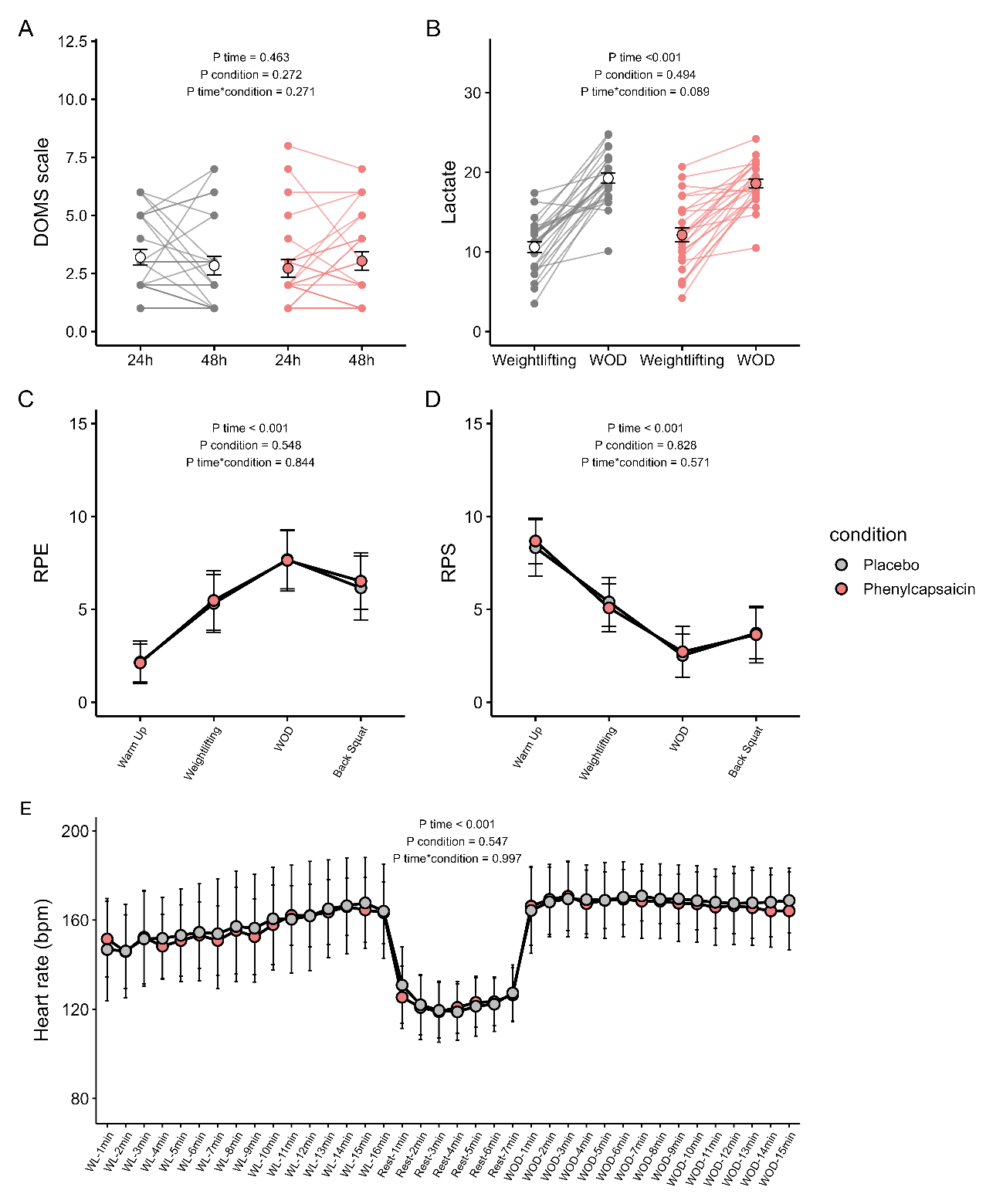


Figure S8. Effects of acute phenylcapsaicin supplementation on subjective and metabolic parameters in men. DOMS scale (A), lactate (B), RPE (C), RPS (D), and heart rate (E) after placebo (grey), or phenylcapsaicin (red) supplementation. Data are depicted as mean ± SD. P values obtained from linear mixed repeated-measures analyses. *Abbreviations*: bpm, beats per minute; RPE, rate of perceived effort; PRS, perceived recovery status.


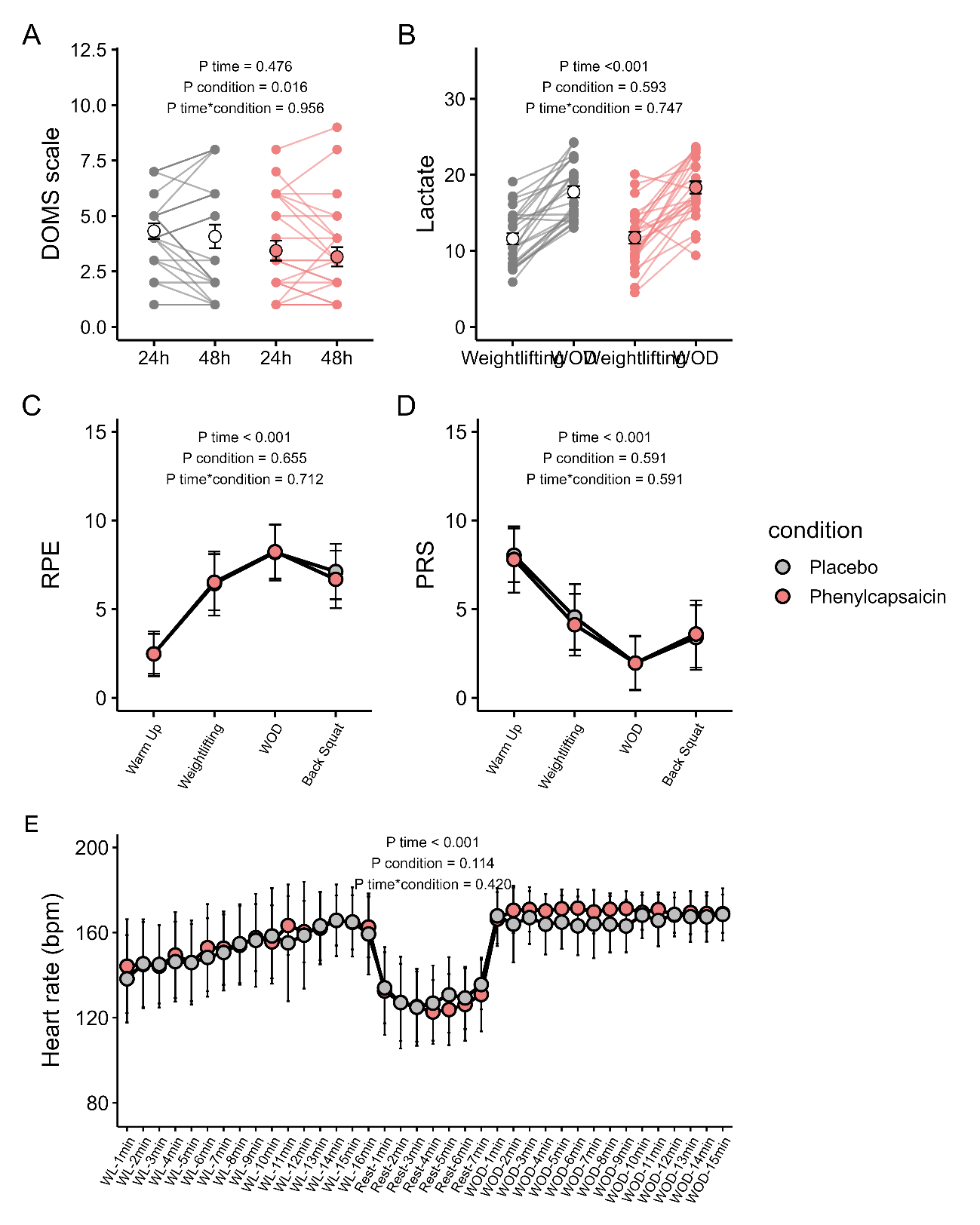


Figure S9. Effects of acute phenylcapsaicin supplementation on subjective and metabolic parameters in women. DOMS scale (A), lactate (B), RPE (C), RPS (D), and heart rate (E) after placebo (grey), or phenylcapsaicin (red) supplementation. Data are depicted as mean ± SD. P values obtained from linear mixed repeated-measures analyses. *Abbreviations*: bpm, beats per minute; RPE, rate of perceived effort; PRS, perceived recovery status.
